# Improvement of the aroma of lily rice wine by using aroma-producing yeast strain *Wickerhamomyces anomalus* HN006

**DOI:** 10.1186/s13568-019-0811-8

**Published:** 2019-06-18

**Authors:** Shoubao Yan, Chen Xiangsong, Xingben Xiang

**Affiliations:** 1 School of Life Science, Huainan Normal University, Huainan, 232001 Anhui People’s Republic of China; 2 Anhui Yingjia Group Co., Ltd., Luan, 237271 Anhui People’s Republic of China; 3 Hefei Institutes of Physical Science, Chinese Academy of Sciences, Hefei, 230031 China; 4 Key Laboratory of Bioresource and Environmental Biotechnology of Anhui Higher Education Institutes, Huainan Normal University, Huainan, 232001 Anhui People’s Republic of China

**Keywords:** Lily rice wine, Aroma-producing yeast, Fermentation, GC–MS analysis, Volatile flavor substances

## Abstract

A fruity aroma-producing yeast strain was isolated with the aim of improving the aroma of fermented lily rice wine, and identified as *Wickerhamomyces anomalus* HN006. In addition, the effects of adding solid residues of previous fermentation cycles, and of fungus α-amylase and *W. anomalus* HN006 supplementation on the biochemical parameters of lily rice wine were evaluated. The optimum quality of the wine, in terms of ethanol and residual sugar content, acidity levels and aroma, was obtained with 5% (w/v) solid residue addition, and supplementation with 10 U/g fungal α-amylase and 2% (v/v) *W. anomalus* inoculum on the 4th day of fermentation. Volatile compound profiles, the total amount of amino acids and the sensory characteristics of the lily rice wines produced by the two fermentation processes were also evaluated and compared. The lily rice wine obtained from our optimized experimental technology produced higher amounts of some esters, free fatty acids, alcohols, aldehydes, ketones, alkenes, volatile phenol and thiazole, in addition to higher total amino acid content and sensory scores compared to the traditionally brewed wine. Our process resulted in an intensification and improvement of lily rice wine aroma.

**Electronic supplementary material:**

The online version of this article (10.1186/s13568-019-0811-8) contains supplementary material, which is available to authorized users.

## Introduction

Lilies are perennial bulbous plants belonging to the monocotyledonous family *Liliaceae*. It has been a valuable medicinal plant in China for centuries, and modern pharmacological studies have shown several benefits of the lily bulb, such as improvement of lung function and cough relief, anti-fatigue and hypoxia tolerance, increase in peripheral white blood cell, protection of gastric mucosa, inhibition of delayed hypersensitivity reaction etc. (Rong et al. 2011). Lily bulbs are rich in nutrients like carbohydrates, proteins, vitamins B_1_, B_2_ and C, magnesium, calcium, iron, potassium, carotene and various alkaloids like colchicines (Gao et al. 2012). Cancer cells treated with colchicine extracted from the lily bulb showed significant in vitro growth inhibition (Chen et al. 2014). Lilies are widely cultivated in China, mainly for food and medicinal purposes, and improvements have been made in recent years in processing the lilies into powder form (Francis et al. 2004), and in the production of compound beverages (Zhu et al. 2014) and soft sweets containing this ingredient (Shang et al. 2016). However, the current processing techniques still have a narrow application range and therefore low economic benefit. Therefore, it is imperative to develop new processing technologies, especially for high value products such as lily rice wine.

Rice wine is one of the most popular alcoholic beverages in China, and has a long history of brewing. With economic globalization, Chinese rice wine has now entered the international market. In addition, the improvement in the standard of living of Chinese people has led to demands of higher quality wine, with additional pro-health effects. Lily bulbs are suitable for rice wine brewing due to their high starch content. Although the process of rice wine brewing retains the nutrients of the lily bulbs, the resulting lily rice wine has suboptimal fragrance and a weak aftertaste. Therefore, it is necessary to improve the aroma in order to promote the rapidly developing lily rice wine industry.

We isolated an aroma-producing yeast strain and used it in brewing lily rice wine, in addition to optimizing the brewing process. The volatile flavor substances obtained from the traditional and modified brewing processes were detected and quantified by solid phase microextraction–gas chromatograph–mass spectrometry (SPME–GC–MS). The sensory characteristics of the final product were also evaluated. Our findings may not only provide a new strategy for enhancing the overall quality of lily rice wine, but also provide a basis for the brewing, quality evaluation and application of other rice wines.

## Materials and methods

### Materials

Dried lilies and debranned polished glutinous rice from the 2016 crop were obtained from the Anhui Yingjia Distillery Group Co. Ltd. (LuAn, Anhui province, China). Wheat *Qu* was purchased from Angel Yeast Co. Ltd. (Yichang, Hubei province, China). Fermented *Zaopei* samples of Chinese strong-flavor liquor was obtained from a brewing plant of Anhui Yingjia Distillery Group Co. Ltd., LuAn, Anhui province. Fungal α-amylase solution was purchased from Shan-dong Longda Bio-Products Co. Ltd. (China). According to the information sheet of the manufacturer, the optimum temperature and pH for fungal α-amylase is in the range of 50–60 °C and 4.0–6.5, respectively. The specific activity of fungal α-amylase is 5000 U/mL, where one unit is defined as the amount of enzyme that hydrolyzes 1 mg of water soluble corn starch per minute under the assay conditions.

### Isolation of aroma-producing yeast strain

Fermented *Zaopei* samples were inoculated into 90 mL yeast extract peptone dextrose medium (YEPD) containing 50 g/L glucose, 5 g/L yeast extract and 10 g/L peptone, in a 250 mL flask. The broth was incubated at 30 °C with constant shaking at 200 rpm for 24–48 h, and the resulting yeast culture was serially diluted with sterile water and spread onto YEPD agar plates. The plates were incubated at 30 °C for 48 h, and the colonies were picked and streaked several times to obtain pure isolates. Each isolated yeast strain was maintained on YEPD agar slants at 4 °C. To further propagate the pure strains, the seed colonies of each were inoculated in the liquid medium and cultured aerobically at 30 °C for 24 h with constant mixing at 200 rpm. The suspension culture was inoculated into 90 mL YEPD at 2% inoculum size (v/v, approximately 1 × 10^7^ CFU/mL), and incubated at 30 °C and 200 rpm for 48 h. Both the liquid and solid cultures were evaluated through smelling (with non-inoculated YEPD agar and liquid medium as control), and the strains producing strong pleasant aroma were selected for further analysis.

### Identification of the aroma-producing yeast strain

The screened strain was identified based on 26S rRNA gene sequencing. Yeast cells at the exponential growth phase were harvested by centrifugation at 12,000 rpm, 4 °C for 10 min and washed with sterile water. Genomic DNA was extracted as described by Gadanho et al. (2003). A fragment of the 26S rRNA gene was amplified by PCR using the forward primer ITS1 (5′-TCCGTAGGTGAACCTGCG-3′) and the reverse primer ITS4 (5′-TCCTCCGCTTATTGATATGC-3′). The 50 µL reaction mix consisted of approximately 100 ng of total DNA, 5 µL 10× buffer, 3 µL 25 mM MgCl_2_, 1 µL 2.5 mM dNTPs, 2 µL of each 10 pM primer and 1 µL 5 U/µL Taq polymerase. The PCR conditions were as follows: initial denaturation at 95 °C for 5 min, followed by 25 cycles of denaturation at 95 °C for 30 s, annealing at 55 °C for 30 s and primer extension at 72 °C for 1 min, followed by a final extension at 72 °C for 10 min. The purified PCR products were then sequenced by Sangon Biotech Company (Shanghai, China). The sequences were submitted to GenBank for BLAST analysis. A phylogenetic tree was constructed by the neighbor-joining method using the MEGA 4.0 program.

#### *W. anomalus* HN006 inoculum preparation for lily rice wine fermentation

The *W. anomalus* HN006 inoculum was prepared in YEPD medium. Pre-cultivation was performed aerobically at 30 °C for 24 h with constant shaking at 200 rpm. The concentration of the yeast cells was adjusted to 1 × 10^7^ cells/mL, and used as the inoculum.

#### Traditional lily rice wine brewing

The traditional method of brewing lily rice wine is shown in Additional file 1. The dried lilies and glutinous rice were mixed at the ratio of 1:4 (w/w), washed, and soaked in 1.5 times the amount of water for about 6–8 h. After soaking, the mixture was cooked with steam for 30 min, and naturally cooled to 30 °C before mixing with wheat *Qu*. The raw material preparation was transferred into a jar and fermented at 30 °C for 7 days.

### Optimization of lily rice wine brewing

The effects of solid residue addition, time of adding fungal α-amylase and *W. anomalus* HN006, and different concentrations of both on the quality parameters of the lily rice wine (ethanol and residual sugar content, acidity levels and aroma) were evaluated. Residue-addition fermentation refers to mixing a portion of solid residues of the previous fermentation batch with the fresh raw material. In the present study, various amounts of the solid residues (0%, 5%, 10%, 15% and 20%, w/w) were added to the soaked raw materials, cooked with steam and then mixed with wheat *Qu* for simultaneous saccharification and fermentation. In addition, fungal α-amylase (10 U/g of raw material) and *W. anomalus* HN006 (2% v/v inoculum corresponding to 1 × 10^7^ CFU/mL) were simultaneously added to the broth, and the effect of adding them at 0, 2, 4 and 6 days of fermentation were analyzed. In addition, different *W. anomalus* HN006 inoculum sizes (2%, 4%, 6% and 8%), and fungal α-amylase concentrations (5 U/g, 10 U/g, 15 U/g and 20 U/g of raw materials) were tested. Fermentation broth samples were taken at regular intervals for analysis, and results represent the averages. All the experiments were performed in triplicates.

### Analysis of the biochemical parameters of lily rice wine

During the fermentation process, broth samples were withdrawn at specific intervals and centrifuged at 5000*g* for 20 min to remove the cellular debris. The concentrations of ethanol, total acids, amino acids and reducing sugars in the supernatant wine were then measured. Ethanol concentration (% v/v at 20 °C) and total acid were determined as described by Yan et al. (2015). Reducing sugar was directly determined by 3,5-dinitro-salicylic acid colorimetry (Miller 1959). Amino acid content was analyzed by HPLC with an ODS HYPERSIL (Agilent Tech Inc., Santa Clara, CA) column and a diode array detector (DAD) at 40 °C with a mobile phase of 20% sodium acetate (0.2 M), 40% acetonitrile and 40% methanol at a flow rate of 1.0 mL/min (Shen et al. 2010).

### Extraction of volatile flavor compounds by HS-SPME

Volatile flavor compounds of lily rice wine were extracted using the headspace solid-phase micro-extraction technique (HS-SPME). The volatile compounds of the fermentation broth were extracted using a 75 mm CAR-PDMS (Supelco, Bellefonte, PA, USA). Each lily rice wine sample (1 mL) was placed in the headspace bottle together with 10 µL of the internal standard 2-octanol (70.2 mg/L in absolute ethanol). The vial was tightly capped, and then equilibrated by ultrasonic vibration in a 60 °C water bath for 10 min. The extraction head was then inserted into the vial and the sample was extracted for 30 min at 60 °C. The extraction head was then introduced into the injection port of the GC–MS system (250 °C for 3 min) and the analytes extracted from the fiber were thermally desorbed.

#### GC-MS analysis

Agilent 6890 GC equipped with an Agilent 5975 mass selective detector (Agilent, USA) was used. Helium was used as the carrier gas at a constant flow rate of 1 mL/min. The temperature of the injector and detector were both set at 250 °C. The analytes were separated through a DB-Wax column (60 m length, 0.25 mm i.d., 0.25 mm film thickness, Agilent) with an oven temperature program of 40 °C (1 min), 5 °C/min to 180 °C (1 min), and 8 °C/min to 230 °C (7 min). The Agilent 5975 MSD was used for identifying the unknown compounds. The electron impact energy was 70 eV, and the ion source and quadruple temperatures were both set at 230 °C. Electron impact (EI) mass spectra was recorded in the 20–550 amu range. All samples were analyzed in triplicates. The mass spectrum data were compared against NIST mass spectral database (Agilent Technologies Inc.) to identify the volatile flavor compounds in lily rice wine samples. The volatile compounds were quantified by comparing their peak areas to that of the 2-octanol internal standard. The amounts of individual constituents present in the sample were calculated and expressed as milligram per liter of wine.

### Sensory evaluation of lily rice wine

The sensory characteristics of taste, smell, aftertaste, acidity and overall acceptability of the final lily rice wine were evaluated by 10 well trained experts working at the Anhui Yingjia Distillery. Four experts were females aged 30–40 years old, and six experts were males and were 28–45 years old. The sensory scores were determined by a 9-point hedonic scale: 1—dislike extremely; 2—dislike very much; 3—dislike moderately; 4—dislike slightly; 5—neither like nor dislike; 6—like slightly; 7—like moderately; 8—like very much; 9—like extremely. The mean intensity scores of all the attributes were calculated and plotted.

### Statistical analysis

The data of volatile compounds was processed using the SPSS version 21.0 statistical package for windows (SPSS Inc., Chicago, IL, USA). Principal component analysis (PCA) was used to investigate the possible differences between the lily rice wine samples.

## Results

### Characterization and identification of aroma-producing yeast strain HN006

Several yeast strains were isolated from fermented *Zaopei*, and after subjecting both their liquid and solid cultures to direct sensorial tests, one strain that produced a strong fruity aroma was selected. The strain was named HN006, and it formed small, spherical, raised, milk-white colonies with no pigmentation on solid medium after 24 h of incubation. The HN006 cells were oval measuring 6 × 10 µm, and multiplied by budding. This aerobic strain grew between 10 and 40 °C with optimum temperature range 28–32 °C, 0–2% NaCl (w/v) with optimum salinity 0–0.5% NaCl (w/v), and pH 6–8 with optimum growth at pH 5. HN006 hydrolyzed casein but not gelatin, starch, Tween 40 and urea, and could utilize glucose, sucrose, maltose, glycerol and d-galactose as sole carbon sources for growth.

**Fig. 1:**
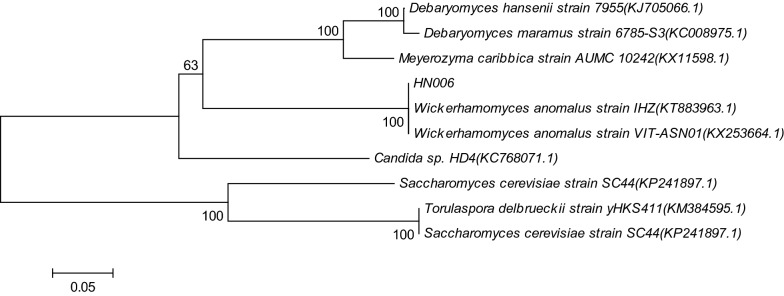
Phylogenetic tree based on the 26S rDNA D1/D2 gene sequences of aroma-producing yeast strain HN006

The D1/D2 domain of 26S rRNA gene is highly conserved and is used for the phylogenetic analysis of higher taxa. The HN006 D1/D2 sequences comprising of 608 nucleotides were submitted to GenBank (http://www.ncbi.nlm.nih.gov), and displayed 100% similarity to that of *Wickerhamomyces anomalus* (GenBank accession KT883963.1 and KX253664.1). A phylogenetic tree was constructed based on the 26S rRNA coding gene sequences of the isolate and the nearest relatives (Fig. 1). Based on the morphological, physiological and molecular data, the fruity-aroma producing strain isolated from *Zaopei* was identified and named as *W. anomalus* HN006.

#### The effect of different amounts of solid residues on the characteristics of lily rice wine

**Fig. 2:**
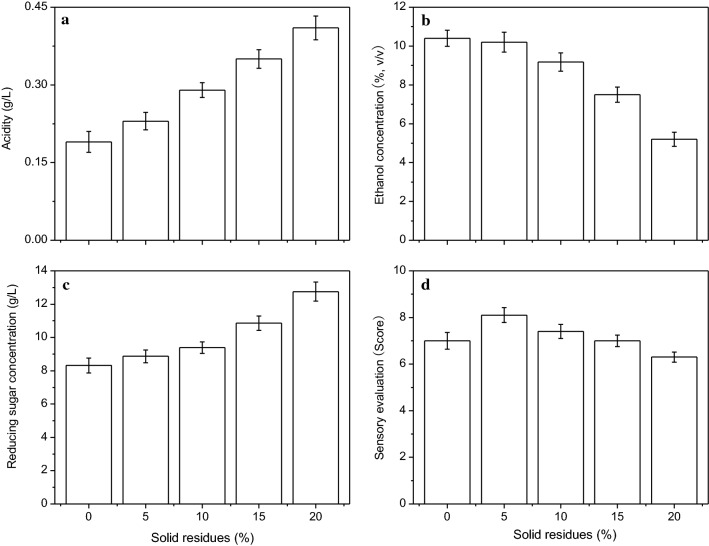
The effects of different amounts of solid residues that obtained from previous turn of fermentation on the acidity (**a**), ethanol concentration (**b**), reducing sugar concentration (**c**), and overall sensory evaluation (**d**) of the lily rice wine

The levels of ethanol, reducing sugars and acidity, and the sensory evaluation scores of the lily rice wine relative to different amounts of solid residues are summarized in Fig. 2. Ethanol concentration remained unchanged as the proportion of solid residues was increased from 0 to 5% (w/v), but decreased significantly at amounts over 5% (w/v). The concentration of reducing sugars gradually increased in the wine with increasing amount of solid residues. This could be due to the presence of acidic substances in the solid residues, which accelerate the hydrolysis of starch in the fermentation media. In addition, a certain level of acidity aids the growth and metabolism of both *Rhizopus* and yeast that are used in rice wine brewing. However, high levels of acidity normally inhibit microbial growth and decreases the efficiency of ethanol fermentation. The acidity levels of the media had expectedly similar trends as that of reducing sugars with increasing solid residue addition. The highest sensory evaluation score was obtained at 5% (w/v) solid residues. Taken together, the optimum amount of solid residues vis-à-vis wine quality was 5% (w/v).

### The effect of the addition time of fungal α-amylase and *W. anomalus* HN006 on lily rice wine quality

The process of hydrolyzing starch into fermentable sugar requires the liquefaction of α-amylase, which converts the starch into dextrin that is subsequently broken down by glucoamylase into fermentable sugars. However, as wheat *Qu* is mainly produced from pure cultures of *Rhizopus* and yeasts in rice flour, its liquefaction power is normally low. To improve the liquefaction efficiency of rice starch, fungal α-amylase (10 U/g raw material) was added to the fermentation broth. In addition, *W. anomalus* HN006 (2% v/v, initial viable cell number 1 × 10^7^ CFU/mL) was also inoculated to improve the sensory properties and ethanol content of lily rice wine, and fermentation was conducted under the optimum conditions mentioned above.

**Fig. 3:**
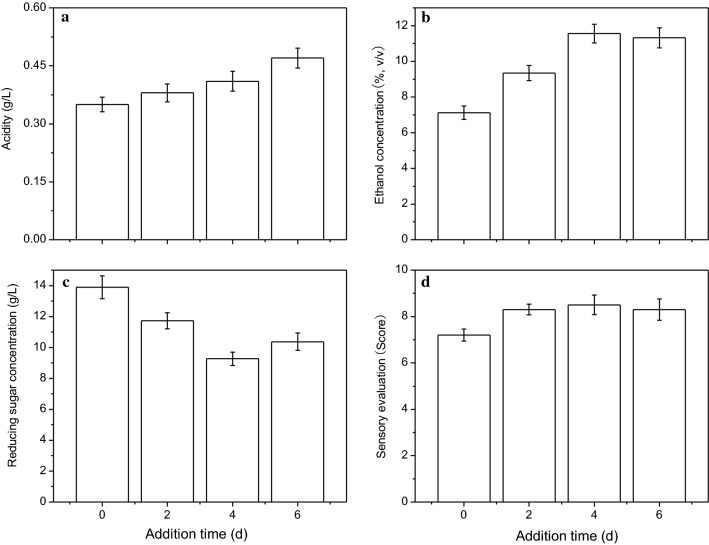
The effect of addition time of fungal α-amylase (10 U/g) and *W. anomalus* HN006 (2%) on the the acidity (**a**), ethanol concentration (**b**), reducing sugar concentration (**c**), and sensory evaluation (**d**) of the obtained lily rice wine

Figure 3 illustrates the effect of the different addition times of fungal α-amylase and *W. anomalus* HN006 on the biochemical parameters of lily rice wine. Both ethanol concentration and sensory evaluation scores were the highest when the amylase and yeast inoculum were added on the 4th day of fermentation, while the reducing sugar content was relatively low for that addition time. In contrast, the addition time had no effect on the acidity level. Therefore, simultaneous addition of fungal α-amylase and *W. anomalus* HN006 on the 4th day of fermentation resulted in optimum quality of the lily rice wine.

Rice wine is mainly produced by the saccharification of *Rhizopus* and the ethanol fermentation of yeast. *Rhizopus* produces glucoamylase which converts starch into glucose, which is partly used for maintaining microbial growth and metabolism, and partly as the raw material for ethanol fermentation. During the initial stages of fermentation, the growth of *Rhizopus* is dominant due to the availability of oxygen and nutrients. With the continuous consumption of oxygen and the accumulation of ethanol and organic acids during fermentation, the growth of *Rhizopus* gradually ceased. The optimum growth of *Rhizopus* in the initial stages led to more efficient saccharification of starch, which increased the ethanol concentration.

The aroma-producing yeasts not only produce aromatic compounds, but also organic acids like acetic acid, which has a strong inhibitory effect on *Rhizopus*. Therefore, the presence of aroma-producing yeast at the initial fermentation stage would inhibit *Rhizopus* growth and reduce starch conversion rate, resulting in low ethanol levels in the wine. On the 4th day of lily rice wine fermentation, the simultaneous addition of fungal α-amylase and *W. anomalus* HN006 not only enhanced the wine flavor but also increased its ethanol concentration. The likely reason is that the large amounts of glucoamylase that had already accumulated in the first 4 days of fermentation rendered any effect of the aroma-producing yeast on glucoamylase production redundant. In addition, the supplementation with fungal α-amylase also promoted the transformation of residual starch into glucose.

### The effect of fungal α-amylase concentration on lily rice wine quality

Alpha amylase (enzyme EC 3.2.1.1) hydrolyzes the α-1,4-glycosidic bond in starch to produce maltose and oligosaccharides. This enzyme is widely used for starch liquefaction in various industries, such as textiles, brewing, baking, detergents, paper etc. The common bacterial α-amylase mainly acts at high temperatures (95–105 °C) and near neutral conditions (pH 5.8–6.8), while fungal α-amylase is suitable for liquefying starch at low temperatures (50–60 °C) and acidic conditions (pH 5–5.5). Therefore, fungal α-amylase is more suitable for lily rice wine brewing.

**Fig. 4:**
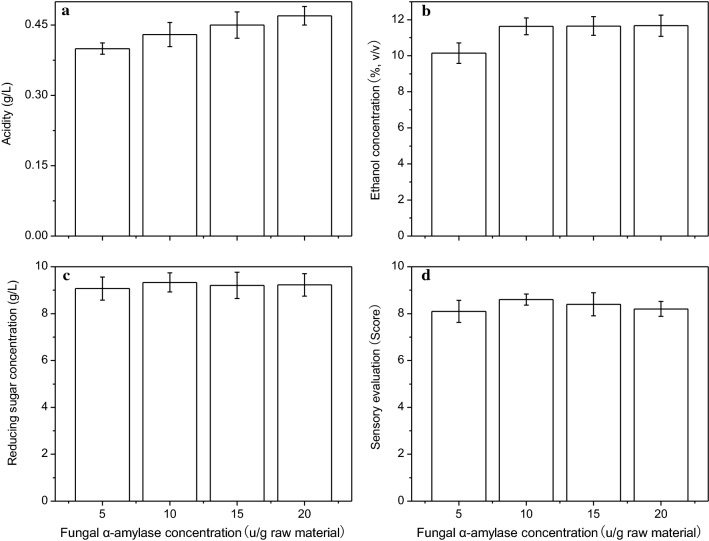
The effect of fungal α-amylase concentration on the the acidity (**a**), ethanol concentration (**b**), reducing sugar concentration (**c**), and overall sensory evaluation (**d**) of the obtained lily rice wine

The effect of fungal α-amylase concentration on lily rice wine fermentation is summarized in Fig. 4. The acidity of the wine increased slightly with increasing concentration of fungal α-amylase, and was not significant. The ethanol concentration increased from 10.35% (v/v) to 11.63% (v/v) as the fungal α-amylase concentration was raised from 5 to 10 U/g of the raw materials, which again was not a significant increase. Similarly, the concentration of reducing sugars also did not change appreciably relative to the fungal amylase. The maximum overall sensory evaluation scores was obtained with the supplementation of 10 U/g fungal α-amylase. Therefore, this concentration of the enzyme was chosen for subsequent fermentation.

### The effect of the inoculum size of *W. anomalus*

**Fig. 5:**
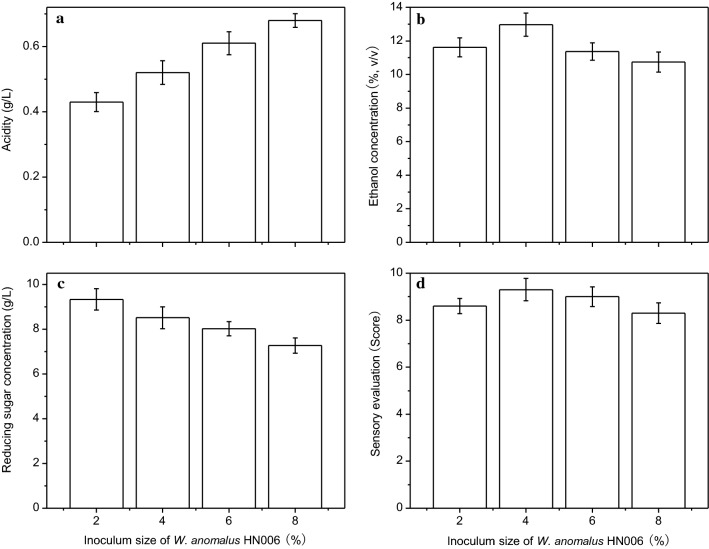
The effect of the inoculum size of *W. anomalus* HN006 on the the acidity (**a**), ethanol concentration (**b**), reducing sugar concentration (**c**), and overall sensory evaluation (**d**) of the obtained lily rice wine

Aroma-producing yeast synthesize volatile flavor substances, mainly consisting of esters and higher alcohols, as part of its metabolism, which give a more harmonious and mellow taste to the wine. Therefore, supplementing the fermentation broth with aroma-producing yeast can improve the flavor of the wine. Figure 5 summarizes the ethanol and reducing sugar content, acidity levels, as well as overall sensory evaluation at different inoculum sizes. The optimum inoculum concentration for maximum ethanol production and overall sensory evaluation was 4% (v/v), and was thus used for all subsequent experiments.

### Volatile flavour compounds in lily rice wine

**Fig. 6:**
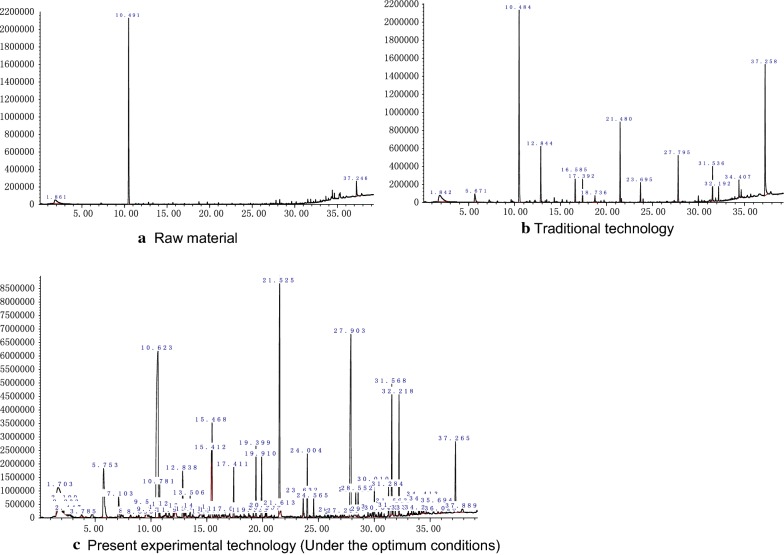
Total ion chromatogram of volatile compounds in the brewing raw material, the lily rice wine that obtained from traditional- and present experimental technology, respectively

In contrast to the raw materials, a variety of volatile components which contribute to the fragrance and the flavor of the wine were detected in the lily rice wine, regardless of the fermentation process. The lily rice wine samples from both fermentation processes were mainly composed of alcohols, acids, esters and alkane compounds (Fig. 6). The wine obtained from traditional fermentation contained 28 volatile flavor compounds, including 11 esters (total content 37.58 mg/L), 8 acids (47.851 mg/L), 4 alcohols (4.743 mg/L), 1 aldehyde (0.214 mg/L), 2 ketones (2.571 mg/L), 1 alkene (1.462 mg/L), and 1 volatile phenol (8.32 mg/L). In contrast, the wine produced by our optimized fermentation process contained 57 volatile compounds, including 21 esters (105.278 mg/L), 14 acids (58.377 mg/L), 10 alcohols (58.266 mg/L), 2 aldehydes (6.007 mg/L), 5 ketones (8.097 mg/L), 3 alkenes (4.976 μmg/L), 1 volatile phenol (12.24 mg/L) and 1 thiazole (1.23 mg/L).

Esters, which impart the fruity and floral odors to Chinese rice wine (Fan and Qian 2006), are formed either by the esterification of alcohols with fatty acids, or through de novo synthesis in the microbial cells by alcohol acetyltransferase using acetyl-CoA and higher alcohols as substrates during fermentation (Mo and Xu 2010). Most aroma-producing yeasts can synthesize esters during fermentation. While only 11 esters were detected in the lily rice wine from traditional brewing, our optimized process resulted in 21 esters under optimal conditions: isopentyl acetate, isoamyl butyrate, ethyl heptanoate, ethyl caprylate, hexyl formate, ethyl nonanoate, ethyl caprate, isoamyl caprylate, phenethyl acetate, ethyl laurate, isopentyl pentadecanoate, ethyl acetate, ethyl myristate, methyl palmitate, ethyl palmitate, ethyl stearate, ethyl oleate, ethyl linoleate, ethyl linolenate etc. (Table 1). In addition, the total amount of esters obtained from the optimized fermentation process was 105.278 mg/L, compared to 37.58 mg/L in the traditional method. Ethyl palmitate was the most abundant ester obtained from the optimized experimental process, accounting for 39.74% of all esters in the lily rice wine. In addition to its influence on the organoleptic characteristics of lily rice wine, ethyl palmitate also imparts a pleasant, fruity fragrance to the wine.

**Table 1:** The volatile aroma compounds identified and quantified in the lily rice wine that obtained from traditional- and present experimental technology (under the optimum conditions)

Number	Aroma compounds	Retention time (min)	Identification	Contents of volatile aroma compounds in 3 samples (mg/L)		
				Raw material	Traditional technology	Present experimental technology
	*Esters*					
ES1	Isopentyl acetate	3.785	MS, RI	ND	ND	0.940
ES2	Isoamyl butyrate	6.775	MS, RI	ND	0.520	0.611
ES3	Ethyl oenanthate	8.163	MS, RI	ND	ND	0.675
ES4	Ethyl caprylate	10.781	MS, RI	ND	1.074	2.481
ES5	Hexyl formate	11.33	MS, RI	ND	0.581	0.673
ES6	Ethyl pelargonate	13.115	MS, RI	ND	ND	0.690
ES7	Ethyl caprate	15.468	MS, RI	ND	1.211	4.380
ES8	Isoamyl caprylate	15.878	MS, RI	ND	ND	0.572
ES9	Phenethyl acetate	19.399	MS, RI	ND	2.145	8.479
ES10	Ethyl laurate	19.91	MS, RI	ND	2.874	6.789
ES11	Isopentyl pentadecanoate	20.257	MS, RI	ND	ND	0.587
ES12	Ethyl acetate	22.87	MS, RI	ND	ND	8.320
ES13	Ethyl myristate	24.004	MS, RI	ND	6.450	7.015
ES14	Methyl palmitate	27.108	MS, RI	ND	ND	0.455
ES15	Ethyl palmitate	27.907	MS, RI	ND	11.625	41.835
ES16	E-11-hexadecenoic acid, ethyl	28.338	MS, RI	ND	ND	2.823
ES17	Ethyl stearate	31.284	MS, RI	ND	ND	2.937
ES18	Ethyl oleate	31.663	MS, RI	ND	0.652	1.758
ES19	Ethyl linoleate	32.218	MS, RI	ND	10.325	12.593
ES20	Ethyl linolenate	33.057	MS, RI	ND	ND	0.336
ES21	Ethyl, 9-cis, 11-trans-octadecadienoate	33.498	MS, RI	ND	0.123	0.329
	Σ			0	37.580	105.278
	*Acids*					
AC1	Acetic acid	11.601	MS, RI	ND	0.65	2.649
AC2	Butanoic acid	15.588	MS, RI	ND	20.349	0.676
AC3	2-Methyl hexanoic acid	16.604	MS, RI	ND	ND	0.712
AC4	Pentadecanoic acid	20.345	MS, RI	ND	ND	0.793
AC5	Octanoic acid	24.565	MS, RI	ND	ND	1.961
AC6	Nonanoic acid	26.534	MS, RI	ND	ND	0.435
AC7	*n*-Decanoic acid	28.552	MS, RI	ND	0.412	3.094
AC8	Dodecanoic acid	31.877	MS, RI	ND	ND	0.726
AC9	Oleic acid	34.243	MS, RI	ND	ND	1.634
AC10	Linoleic acid	36.066	MS, RI	ND	2.11	10.16
AC11	Myristic acid	34.413	MS, RI	ND	1.418	2.24
AC12	Pentadecanoic acid	35.694	MS, RI	ND	0.354	1.235
AC13	Hexadecanoic acid	37.246	MS, RI	6.77	0.213	0.242
AC14	*n*-Hexadecanoic acid	37.265	MS, RI	ND	22.345	31.820
	Σ			6.77	47.851	58.377
	*Alcohols*					
AL1	Isoamyl alcohol	7.254	MS, RI	ND	0.714	5.331
AL2	2-Nonanol	9.519	MS, RI	ND	1.27	2.186
AL3	2,3-Butanediol	13.506	MS, RI	ND	0.390	2.373
AL4	1-Octanol	13.746	MS, RI	ND	ND	0.664
AL5	2-(2-Ethoxyethoxy)-ethanol	15.184	MS, RI	ND	ND	0.482
AL6	2-Tetradecanol	17.411	MS, RI	ND	ND	5.858
AL7	Phenylethyl alcohol	21.525	MS, RI	ND	2.369	39.518
AL8	2-Dodecanol	21.613	MS, RI	ND	ND	0.561
AL9	Isoborneol	27.291	MS, RI	ND	ND	0.625
AL10	Glycerin	29.429	MS, RI	ND	ND	0.668
	Σ			0	4.743	58.266
	*Aldehydes*					
AD1	Decanal	12.131	MS, RI	ND	0.214	0.683
AD2	4-Methylbenzaldehyde	23.632	MS, RI	ND	ND	5.324
	Σ			0	0.214	6.007
	*Ketones*					
KE1	2-Octanone	7.103	MS, RI	ND	0.221	2.529
KE2	2-Undecanone	14.472	MS, RI	ND	ND	0.391
KE3	2-Tridecanone	19.159	MS, RI	ND	ND	0.473
KE4	2-Nonanone	23.695	MS, RI	ND	2.350	0.67
KE5	5-Heptyldihydro-2(3*H*)-furanone	30.01	MS, RI	ND	ND	4.034
	Σ			0	2.571	8.097
	*Alkenes*					
AK1	1,3,5-(3*E*,5*Z*)-1,3,5-Undecatriene	9.778	MS, RI	ND	ND	0.623
AK2	2-Tetradecene	30.167	MS, RI	ND	1.462	0.651
AK3	1,15-Hexadecadiene	30.634	MS, RI	ND	ND	3.702
	Σ			0	1.462	4.976
	*Volatile phenols*					
VP1	4-Vinylguaiacol	26.698	MS, RI	ND	8.32	12.24
	Σ			0	8.32	12.24
	*Thiazoles*					
TH1	2-Mercapto-4-phenylthiazole	12.11	MS, RI	ND	ND	1.23
	Σ			0	0	1.23

*MS* compounds were identified by MS spectra, *RI* compounds were identified by comparison with RI from literature, *ND* not detected

Acids constitute a fairly large group of volatile compounds in rice wine, whose content and types can influence the quality of the final product. An appropriate amount of organic acids inhibit the growth of contaminating bacteria, and improve the mellow aftertaste and the flavor of the wine. The organic acids can also promote the formation of esters during fermentation. The lily rice wine obtained from our optimized fermentation process had higher content of different organic acids, except that of butanoic acid, compared to that of traditionally brewed wine. Acetic acid, butanoic acid, *n*-decanoic acid, linoleic acid, myristic acid, pentadecanoic acid, hexadecanoic acid and *n*-hexadecanoic acid were identified in the two lily rice wine samples, of which *n*-hexadecanoic acid, a product of lipid oxidation, was the most abundant in both. Acetic acid affects the aroma and flavor of wines due to its pungent smell (Soufleros et al. 2004). However, only 0.65 mg/L and 2.649 mg/L acetic acid was detected in the final lily rice wine product obtained from traditional- and modified fermentation respectively. This contradicts the observation that acetic acid is the dominant organic acid in alcoholic beverages (Satora et al. 2008). The low concentration of acetic acid in the rice wines could be due to its transformation into fatty acids during the fermentation process. Two unsaturated fatty acids, oleic acid and linoleic acid, were detected in the rice wine obtained from the optimized fermentation process (Table 1), which contributed to the soapy texture, green color and fatty odor of the wine.

Higher alcohols are among the most important flavor compounds in rice wine and contribute to the aroma and clean-taste of the wine. The higher alcohols are predominantly formed by the yeast from α-keto acids, either via the degradation of amino acids (valine, leucine, isoleucine, threonine, phenylalanine) by the Ehrlich pathway or de novo biosynthesis from the carbon source. In this study, the main higher alcohols identified in the lily rice wine obtained from the optimized fermentation were isoamyl alcohol (5.331 mg/L), 2-nonanol (2.186 mg/L), 2,3-butanediol (2.373 mg/L), 1-octanol (0.664 mg/L), 2-(2-ethoxyethoxy)-ethanol (0.482 mg/L), 2-tetradecanol (5.858 mg/L), phenylethyl alcohol (39.518 mg/L), 2-dodecanol (0.561 mg/L), isoborneol (0.625 mg/L), and glycerin (0.668 mg/L). Isoamyl alcohol, 2-nonanol, 2,3-butanediol and phenylethyl alcohol were the main higher alcohols detected in both lily rice wine samples, but their amounts were significantly lower in the traditionally brewed wine (Table 1). Isoamyl alcohol imparts a strong banana flavor, and also reduces the amount of bitter-tasting amino acids like leucine which improves the wine taste. Isobutanol has a faint smell of allyl alcohol with bitter notes, while 2,3-butanediol has a unique bitter taste and a buttery odor note (Bartowsky and Henschke 2004). Phenylethyl alcohol, which contributes to rosy and honey aroma, is usually present in high concentration in Chinese rice wines (Fan and Qian 2006).

Two aldehydes and five ketones were identified in the lily rice wine obtained from the optimized fermentation, with total amounts of 6.007 mg/L and 14.104 mg/L respectively, both of which were higher than that in the traditionally brewed wine. The most potentially important aldehyde was 4-methyl benzaldehyde, which imparts a bitter almond aroma. It could be formed by the oxidation of benzyl alcohol, by the microbial decomposition of aromatic amino acids, or from phenyl acetic acid. Furthermore, aldehydes and ketones might also be the oxidation products of linoleic acid (Liu et al. 2015).

Three alkenes (1,3,5-(3*E*,5*Z*)-1,3,5-undecatriene, 2-tetradecene, and 1,15-hexadecadiene) were detected in the lily rice wine obtained from our modified fermentation process, while only one alkene was present in the traditionally fermented rice wine. The contribution of the alkenes to rice wine aroma and flavor are not clear so far. One volatile phenol (4-vinylguaiacol) was detected in both lily rice wines samples (Table 1), and imparts spicy, smoky, and clove-like odors. The volatile phenols detected in the samples can originate from *p*-coumaric and ferulic acids by enzymatic or thermal decarboxylation (Ye et al. 2014). In addition, low amounts of one thiazole (2-mercapto-4-phenylthiazole) was identified in the lily rice wine obtained from the optimized fermentation, but its contribution to rice wine aroma and flavor needs further investigation.

### Amino acids content of lily rice wine

Amino acids are not only essential for yeast nutrition, but are also the precursors of the aroma compounds in Chinese rice wine, and therefore an important quality parameter (Shen et al. 2010). Amino acids are mainly derived from the enzymatic degradation of proteins in the raw material by the wheat *Qu* proteases, as well as autolysis of yeast and other microbes. During fermentation, some amino acids are used by yeast as nutrients, some are transformed into higher alcohols, and the remaining become part of the rice wine (Ye et al. 2014). The amino acid content in the lily rice wine samples are summarized in Table 2. Seventeen amino acids were identified, the total amount and that of each amino acid was higher in the rice wine obtained from optimized fermentation process (total content 4282.6 ± 40.26 mg/L) compared to the traditionally brewed wine (total content 2777.4 ± 38.9 mg/L), with an increase of 54.2%. This may be due to the increased acidity in our modified process which helps in protein digestion. Furthermore, some organic acids that are produced by the aroma-producing yeast may accelerate microbial autolysis.

**Table 2:** Contents of amino acids in the lily rice wine that obtained from traditional- and present experimental technology (under the optimum conditions) (mg/L)

Amino acid	Present experimental technology (mg/L)	Traditional technology (mg/L)	Amino acid	Present experimental technology (mg/L)	Traditional technology (mg/L)
Asp	290.5 ± 10.4	177.8 ± 12.1	Cys	54.2 ± 3.2	36.7 ± 2.1
Glu	659.2 ± 11.3	393.4 ± 7.8	Val	388.6 ± 8.9	250.3 ± 9.2
Ser	38.2 ± 3.1	11.3 ± 2.2	Met	142.9 ± 10.5	93.6 ± 8.3
His	23.7 ± 2.3	11.2 ± 1.8	Phe	329.4 ± 12.8	267.6 ± 10.3
Gly	253.7 ± 5.4	121.2 ± 8.2	Ile	242.6 ± 9.9	127.9 ± 10.2
Thr	225.9 ± 6.8	118.3 ± 5.5	Leu	473.8 ± 11.2	342.6 ± 9.7
Arg	497.3 ± 7.2	400.6 ± 7.4	Lys	279.1 ± 13.1	217.8 ± 9.3
Tyr	383.5 ± 6.3	207.1 ± 8.2	Sum of amino acid	4282.6 ± 40.26	2777.4 ± 38.9

## Discussion

In present study, the volatile flavor substances in the wines obtained from the traditional and novel brewing technologies were identified by SPME–GC–MS. The lily rice wine obtained from the optimized fermentation process had higher amounts of some esters, free fatty acids, alcohols, aldehydes, ketones, alkenes, volatile phenol and thiazole, in addition to higher total amino acid content compared to the traditionally brewed wine. The optimized fermentation process resulted in an intensification and improvement of lily rice wine aroma.

**Fig. 7:**
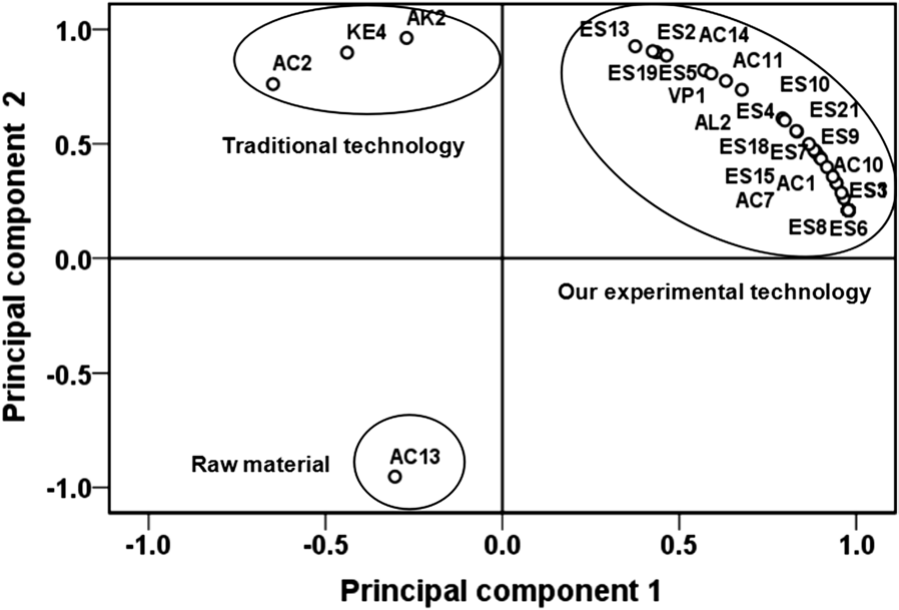
Principal component analysis of 57 volatile compounds from the samples of the lily rice wine that obtained from traditional- and present experimental technology, respectively. Each number was coincided with the specific volatile compound which is shown in Table 1

Additionally, SPSS version 21.0 was used to analyze the data of volatile compounds of the brewing raw materials and the two lily rice wine samples by the principal component analysis (PCA), and the results are shown in Fig. 7. A two dimensional analysis of the principal components was attempted using the component data matrix shown in Table 1.

The first and second principal components accounted for 92.5% and 3.79% of the variance respectively. The first principle component of the lily rice wine obtained from the optimized fermentation was located on the positive side of the factor, and characterized mainly by esters, fatty acids and the volatile phenol 4-vinylguaiacol. The esters were isoamyl butyrate, ethyl heptanoate, ethyl caprylate, hexyl formate, ethyl nonanoate, ethyl caprate, phenethyl acetate, ethyl laurate, ethyl palmitate, ethyl oleate, ethyl linoleate, ethyl and 9-cis, 11-trans-octadecadienoate. The fatty acids were acetic acid, *n*-decanoic acid, linoleic acid and myristic acid. In contrast, the principal component of the traditionally brewed lily wine was on the negative side of the factor, distinguished by the production of butanoic acid, alkene 2-tetradecene and ketone 2-nonanone. Hexadecanoic acid that originated from fatty acids was also found on the negative region of the factor.

PCA results indicated that the two lily rice wine samples correlating with particular groups of volatile compounds were clearly discriminated in the loading plot (Fig. 7), and the wine obtained from the optimized fermentation process had a long distance with the traditionally produced wine and the raw materials. The main flavor components of the lily rice wine obtained from the modified process were isoamyl butyrate, ethyl oenanthate, ethyl caprylate, hexyl formate, ethyl pelargonate, ethyl caprate, phenethyl acetate, ethyl laurate, ethyl palmitate, ethyl oleate, ethyl linoleate, ethyl, 9-cis, 11-trans-octadecadienoate, acetic acid, *n*-decanoic acid, linoleic acid, myristic acid and 4-vinylguaiacol, which were clearly distinguishable from those of the traditional wine and raw materials. The lily rice wine obtained from traditional brewing was located on the positive semi axle region of the second principle component and far from other samples. The main flavor components distinguishing the traditionally brewed lily rice wine were butanoic acid, 2-tetradecene and 2-nonanone. Hexadecanoic acid was the main flavor component that distinguished raw materials from the lily rice wine samples. These observations demonstrated the relationship between the volatile compounds and the fermentation process.

**Fig. 8:**
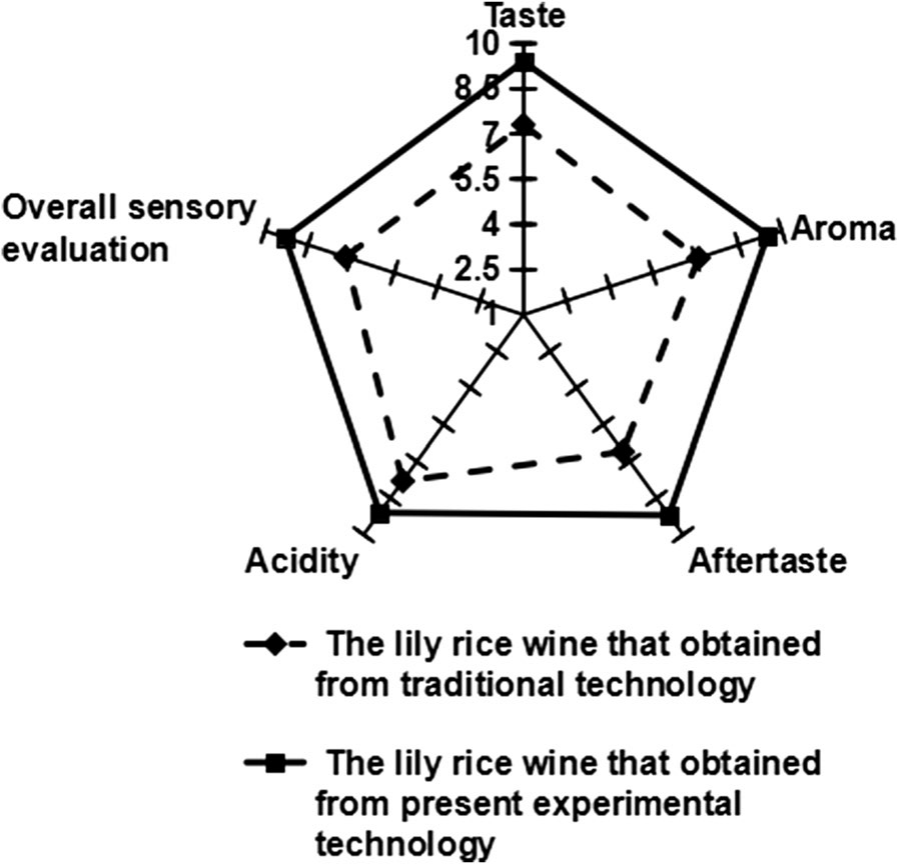
Spider diagram of descriptive sensory analysis for the lily rice wine that obtained from traditional- and present experimental technology

The sensory evaluation of lily rice wine was also investigated (Fig. 8) and the lily rice wine obtained from the optimized fermentation process scored better in all quality parameters such as acidity, taste, aroma, aftertaste and overall sensory evaluation, increasing specifically by 1.3, 2.4, 2.1, 2.6 and 1.5 points respectively compared to the traditionally brewed wine. This could be due to the higher concentration of esters, especially ethyl palmitate, in the lily rice wine brewed by the modified process. In addition, high concentrations of fatty acids, such as *n*-hexadecanoic acid, pentadecanoic acid, linoleic acid, myristic acid etc., may also contribute to the enhanced flavor and taste of the wine. A previous study has reported that organic acids harmonize the flavor and taste of wine and increase its softness (Ye et al. 2014). Taken together, sensory evaluation indicate better quality of the wine obtained by the optimized fermentation.

Volatile aroma compounds play an important role in the quality of rice wine, and are influenced by factors such as raw materials, wheat *Qu* and the fermenting microbes, especially yeast. During rice wine brewing, yeast transforms the fermentable sugars into ethanol and carbon dioxide, along with producing higher alcohols, acids, esters, aldehydes etc., which greatly influence the wine aroma. Different yeast species produce different kinds and amounts of volatile substances, which contributes to the different flavors. Therefore, it is necessary to screen new, aroma-producing yeast strains to increase the levels of volatile flavor substances in rice wine. Addition of the aroma-producing yeast strain of *W. anomalus* HN006 resulted in significantly higher amounts of aroma substances, a more complex volatile profile, and higher sensory scores than those obtained using traditional rice wine brewing methods. This strain can be tested in starters or adjunct cultures of other types of rice wine as well.

## Additional file

**Additional file 1.** Flow diagram of lily rice wine production.

## Data Availability

Please contact author for data requests.

## Abbreviations

SPME–GC–MS: solid phase microextraction–gas chromatograph–mass spectrometry
YEPD: yeast extract peptone dextrose medium
HS-SPME: headspace solid-phase micro-extraction technique
PCA: principal component analysis
