# Supplementary figures and images for: Improvement of the aroma of lily rice wine by using aroma-producing yeast strain Wickerhamomyces anomalus HN006
Source: AMB Express. 2019 Jun 18;9:89. doi: 10.1186/s13568-019-0811-8 (PMC6582127; doi:10.1186/s13568-019-0811-8)

**Additional file 1**


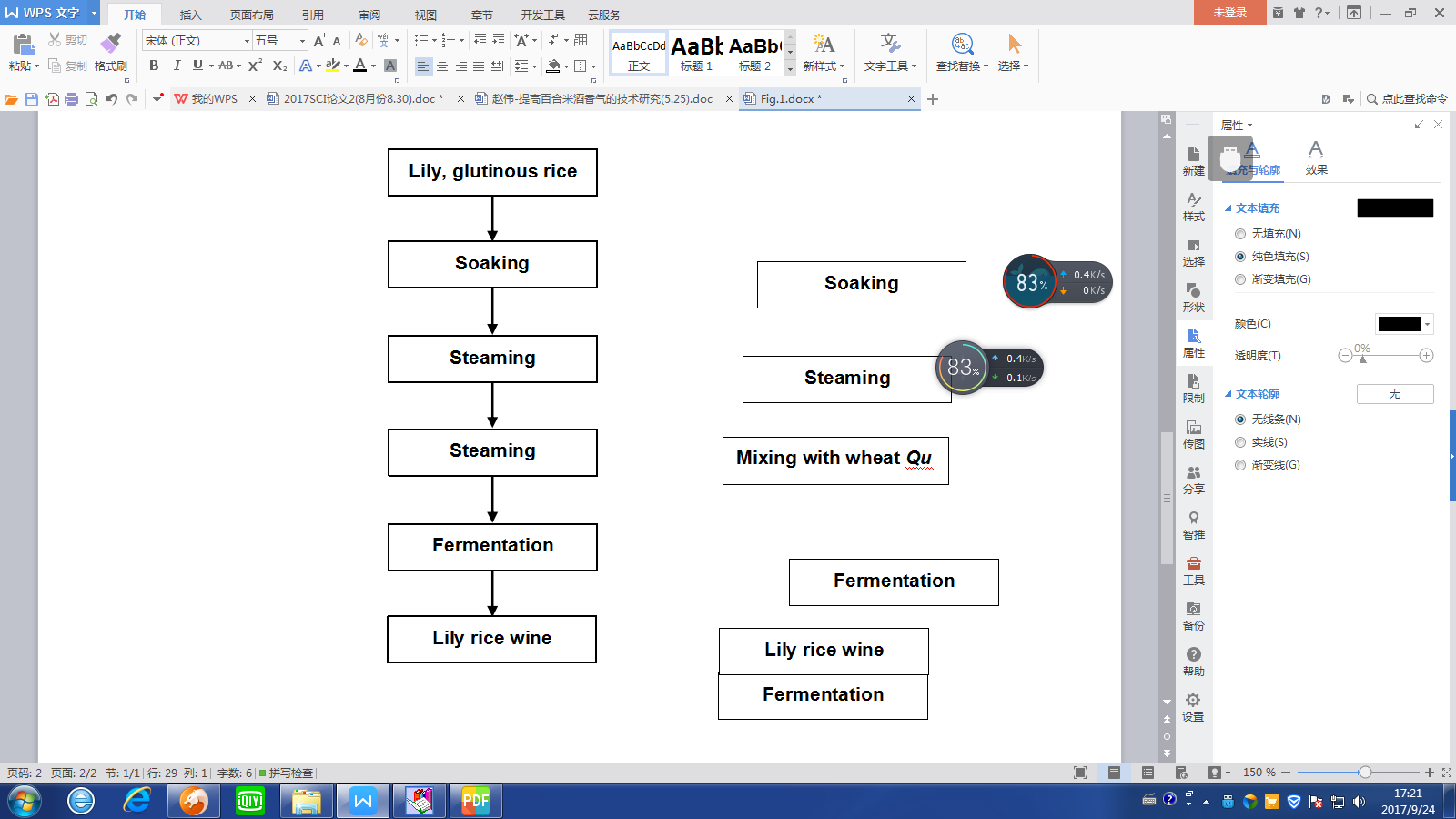


Flow diagram of lily rice wine production

Supplement: Supplementary file 1 — Additional file 1. Flow diagram of lily rice wine production. [file 13568_2019_811_MOESM1_ESM.doc]
